# Update and ADMET Profile of the Latin American Natural Product Database: LANaPDB

**DOI:** 10.1002/minf.70013

**Published:** 2025-12-10

**Authors:** Alejandro Gómez‐García, Martin J. Lavecchia, Dionisio A. Olmedo, Pablo N. Solís, José L. Medina‐Franco

**Affiliations:** ^1^ DIFACQUIM Research Group Department of Pharmacy School of Chemistry Universidad Nacional Autónoma de México Mexico City Mexico; ^2^ CEQUINOR (UNLP‐CONICET, CCT La Plata, associated with CIC PBA) Departamento de Química Facultad de Ciencias Exactas Universidad Nacional de la Plata La Plata Argentina; ^3^ Center for Pharmacognostic Research on Panamanian Flora (CIFLORPAN) College of Pharmacy University of Panama Panama City Panama

**Keywords:** ADMET, databases, LANaPDB, natural products, open‐access

## Abstract

For more than 5 years, several countries in Latin America have been developing and updating compound databases of natural products (NPs) isolated and characterized by their countries. In parallel, multiple research groups have been collaborating and assembling a unified Latin American Natural Product Database (LANaPDB), an open‐access compound collection representative of Latin America that stands out as a geographical region distinct from its vastness and richness of NP resources. Herein, we report a significant update of LANaPDB, which gathers NPs from eight countries. Major updates to the database include adding 1,164 new compounds obtained from NaturAr, a NP collection from Argentina published in 2025, and 132 new compounds from Panama. The updated LANaPDB has 14,742 nonduplicate compounds. Moreover, a comprehensive evaluation of 41 ADMET (absorption, distribution, metabolism, excretion, and toxicity)‐related parameters was carried out for LANaPDB, and the results were compared with one of the largest NP databases, the Universal Natural Product Database, and the approved small‐molecule drugs. The results indicated that the three databases have a very similar ADMET profile. Besides, most of the LANaPDB compounds presented high bioavailability, volume of distribution, plasma protein binding rate, blood–brain barrier penetration, susceptibility to CYP3A4, and half‐life less than 12 h. Moreover, most of the LANaPDB compounds were predicted with a low probability of inducing toxicity‐related reactions. The third version of LANaPDB and the codes for the curation and determination of 41 ADMET‐related parameters are freely available at https://doi.org/10.5281/zenodo.15595030. The code is general and can be used to analyze other compound libraries.

## Graphical abstract

LANaPDB was updated with 1,164 nonduplicate new compounds coming from Argentina and Panama. Moreover, 41 ADMET (absorption, distribution, metabolism, excretion, and toxicity)‐related parameters were determined, and the results were compared with the Universal Natural Products Database and the approved small‐molecule drugs. The results indicated that the three databases have a very similar ADMET profile.

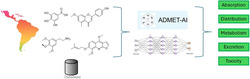

## 1 Introduction

Historically, natural products (NPs) have been an essential source of bioactive molecules with therapeutic activity. Of all the approved drugs, not just the approved by the Food and Drug Administration (FDA), from 1981 to 2019, 49.4% are NPs and NP derivatives (18.9% corresponded to semisynthetic NPs, 3.2% synthetic compounds with a NP pharmacophore, 11.5% synthetic compounds that mimics NPs, 11% synthetic compounds with a NP pharmacophore and mimics NPs, 3.8% to NPs and 0.8% to NPs botanical which are defined as a mixture) [1]. The percentage of NPs and NP derivatives rises to 66.8% when the biologicals and vaccines are not taken into account, and the comparison is made with the remaining group that are the synthetic molecules which are not related to NPs. The identification and development of new drug candidates from NPs using computer‐aided drug design (CADD) techniques has become more relevant, due to the continuous advances in the chemoinformatics and artificial intelligence (AI) [2, 3, 4]. Furthermore, from 1981 to 2019, the discovery process of more than 70 commercialized drugs included some form of computational technique [5]. Moreover, CADD NP‐based represented a main approach in the design and identification of lead compounds during the severe acute respiratory syndrome coronavirus 2 (SARS‐CoV‐2) pandemic outbreak [6, 7, 8, 9].

NP databases play a crucial role during the CADD process, they can be used to identify bioactive molecules with therapeutic applications employing virtual screening or AI methodologies [3, 4, 10, 11, 12]. Moreover, NP databases are invaluable sources of structural molecular fragments whose unprecedent combinations have been used for the discovery of new bioactive molecules with unexpected or novel bioactivities which are known as pseudonatural products. In fact, one‐third of historically developed bioactive molecules and of currently commercially available screening compounds are pseudonatural products. Besides, 63% of the scaffolds in recent clinical compounds are made up of 176 NP fragments [13]. During the early stages in the CADD, it is of utmost importance the assessment of the absorption, distribution, metabolism, excretion, and toxicity (ADMET) profile in compound databases to identify those molecules with a desirable pharmacokinetic profile in order to raise the probability to succeed in the later drug development stages. In the clinical phases I, II, and III, over 75% of the drug candidates fail to pass to the next phase [14, 15], absorption, distribution, metabolism, and excretion properties are related to the failure of 40% of the candidates, and toxicity is responsible in 30% of the cases [14, 16]. Early assessment of ADMET properties can minimize the time and cost of screening and testing by identifying the strongest candidates for development and rejecting those with a low probability of success [17].

Up to 2023, there have been reported eighteen free online servers that allow the prediction of the ADMET profile and have already been assessed, comparing their advantages and disadvantages [18]. By mid‐2024 was published ADMET‐AI, a free tool for the prediction of the ADMET profile [19]. The predictions can be made in their online website, nonetheless, it stands out for its capacity to carry out the calculations locally. When working with large compound databases that have thousands of compounds it is not feasible to make the ADMET calculations in the available free online servers because they just support a limited amount of compounds, usually, up to one thousand chemical structures. Besides, with the appropriate hardware, local calculations can be performed faster than those processed by free online ADMET servers. That is why it stands out the feature of ADMET‐AI to perform the calculations locally, allowing the users with large databases (thousands of compounds) to perform the calculations of the compounds all at once in a reasonable amount of time. Furthermore, compared to other servers that predict the ADMET profile, ADMET‐AI has shown the best average performance, accurately predicting the experimental values of 22 ADMET parameters [19]. Moreover, ADMET‐AI aligns with key success factors for effective toxicity prediction outlined in mid‐2025 [20]. It leverages curated datasets, robust machine learning algorithms, and rigorous validation strategies to ensure reliable ADMET predictions. By assessing a wide range of pharmacokinetic and toxicological properties, and offering high predictive performance, ADMET‐AI meets practical demands for large‐scale compound evaluation [19].

Among the largest open‐access NP databases that can be employed in CADD and other chemoinformatics applications are COCONUT 2.0 (695,130 molecules) [21], SuperNatural 3.0 (449,058, molecules) [22], and the Universal Natural Products Database (UNPDB) (∼229,000 molecules) [23, 24]. Moreover, there are other NP databases that can be used in the CADD process that contain NPs isolated and characterized in certain geographical areas. To mention some representative examples, TCMBank (61,966 molecules) is the largest database of NPs from traditional Chinese medicine [25], ANPDB (∼6500 molecules) is the most extensive collection of NPs from Africa [26], and IMPPAT (∼10,000 molecules) the most comprehensive NPs database from India [27]. Another major open‐access and curated database that can be used in CADD is LANaPDB (14,742 molecules in the 3° version) which is the major collection of NPs isolated and characterized in Latin America, it gathered and standardized eleven Latin American NP databases from eight different countries [28, 29, 30]. It is estimated that Latin America is home to at least a third of global biodiversity [31]. Therefore, Latin America is an invaluable source of bioactive molecules, many of them contained in LANaPDB. Since the release of the first two versions of LANaPDB, rapid and new developments in NPs in Latin America have occurred.

Here, we report the third comprehensive update of LANaPDB, a database that aims to gather and standardize the information of all the Latin American NP databases. We also discuss the insights of a comprehensive characterization of the ADMET profile of LANaPDB based on the recent platform ADMET‐AI which algorithms use AI. The ADMET profile of the two previous versions of LANaPDB had not been reported before. The updated database with 14,742 unique compounds and the project code is freely available at https://zenodo.org/records/15595031.

## 2 Methods

We employed the Python programming language, version 3.12.5. Moreover, the following Python packages: RDKit (2024.3.5) [32], MolVS (0.1.1) [33], ADMET‐AI (1.3.1) [19], and seaborn (0.13.2) [34] were used.

### 2.1 LANaPDB Update and Data Curation

The second version of LANaPDB was updated to the third version, adding 1,164 new compounds obtained from two different databases: NAPROPMA, previously known as CIFPMA [35, 36] and NaturAr [37]. The curation process was made in the Python programming language, employing the RDKit and MolVS packages. The function and package associated with every step or steps is in parentheses. From the SMILES strings [38] the molecules are converted into an object that can be handled by RDKit and MolVS for the chemical structures manipulation (MolFromSmiles‐RDKit). The aromatic atoms are identified and kekulized. This step is necessary for the correct functioning of the algorithm that carries out the next step that is the valency verification. It is verified for the individual atoms in the molecules if they have a correct valency, the molecules with incorrect valency atoms are rejected. The kekulized aromatic atoms are changed to the aromatic representation, that is, SMILES strings for benzene: kekulized ‘C1=CC = CC = C1’, aromatic ‘c1ccccc1.’ Moreover, it is determined which bonds are conjugated and the hybridization of each atom. The labeling of the conjugated atoms and their hybridization does not appear in the final output SMILES, but is essential for the proper functioning of most of the RDKit and MolVS functions. All the above steps are executed sequentially with just one function (SanitizeMol‐RDKit, this function can be executed from the MolFromSmiles function if the argument sanitize=True which by default is true). Afterward, the explicit hydrogen atoms are removed (i.e., SMILES strings of methanol with explicit ‘[H]C([H])([H])[O][H]’ and implicit hydrogens ‘CO’), since some RDKit functions trouble with the explicit hydrogen representation (RemoveHs‐RDKit). The metal atoms that are covalently bonded to nonmetals are disconnected (disconnect‐MolVS). From the molecules that used to be connected with metals or other salts, just the largest fragment is kept (choose‐MolVS). When applicable, one or more transformations (there are 22 transformation rules that can be consulted in the MolVS documentation [33]) are applied to correct common drawing errors and correctly represent the functional groups with their correct charge (normalize‐MolVS). In molecules that are partially ionized, the strongest acid is protonated first (reionize‐MolVS). The remaining molecules that can be neutralized are neutralized (uncharge‐MolVS). The stereochemistry is reassigned to ensure the correct label of chiral centers and double bond configurations without altering the original valid stereochemicalinformation (AssignStereochemistry‐RDKit). This last step tries to address some of the identified stereochemistry inconsistencies that have been found in the major freely available databases of chemical compounds [39, 40]. The canonical RDKit‐SMILES strings are obtained for all the molecules (MolToSmiles‐RDKit). From the canonical RDKit‐SMILES strings, the duplicated structures are removed. Errors or issues with molecules that cannot be corrected automatically are detected and highlighted (validate_smiles‐MolVS) to be manually corrected or if it is the case the molecule is eliminated. Moreover, are highlighted the molecules that contain different elements than C, H, O, N, P, S, F, Cl, Br, I, B, Si, and Se to manual remotion.

The ADMET profile of LANaPDB was compared with two reference databases: the approved drugs database version 5.1.12 (released by DrugBank in March 2024) [41] and the UNPDB which is one of the largest open‐access NP databases [23, 24]. The approved drugs database version 5.1.12 is referred to in this text as ’approved small‐molecule drugs’, since only drugs from that category were retained at the end of the curation process. Both datasets underwent the same curation process of LANaPDB described above. Additionally, from the approved small‐molecule drugs were removed the drugs within its classification included the label experimental, investigational, withdrawn, illicit or nutraceutical. The drugs labeled as ’veterinary approved’ were retained because most of them were initially approved for human use, and many are still widely employed in human medicine. In many cases, the veterinary applications were approved subsequently.

### 2.2 ADMET Profile

For LANaPDB, 41 parameters of the ADMET profile were calculated employing the Python programming language with the ADMET‐AI package and compared with the reference databases. With the Python programming language with the seaborn package were constructed violin and box plots to describe the distribution of the calculated ADMET parameters.

## 3 Results and Discussion

### 3.1 LANaPDB Update and Data Curation

LANaPDB was updated from its second version (13,578 compounds) to the third version (14,742 compounds), integrating a total of 1,164 new compounds, 132 NPs coming from the latest update of NAPROPMA [35, 36] which is a NP database from Panama and 1,032 compounds from NaturAr [37]. NaturAr is a newly generated and curated database that was integrated into LANaPDB Table 1. It is a freely available database of NPs isolated and characterized in Argentina and can be consulted and downloaded from its official website (https://naturar.quimica.unlp.edu.ar/es/). With the inclusion of NaturAr, the NPs of Argentina (1,032 compounds) are the third most abundant NPs in LANaPDB, after Brazil (10,599 compounds) and Mexico (1,440 compounds). For the LANaPDB update, 168 compounds were initially considered from the latest update of NAPROPMA and 1,278 compounds from NaturAr. Nonetheless, after the curation process that includes the elimination of duplicated molecules, only 132 compounds remained of NAPROPMA and 1,032 compounds from NaturAr.

**TABLE 1:** Natural product databases in the updated version of LANaPDB.

Database	Number of compounds	Source	General description	Reference
NaturAr (Argentina)	1278	Plants Animals Fungi	Natural products isolated and characterized in Argentina.	[37]
NuBBE_DB_ (Brazil)	2223	Plants Microorganisms Terrestrial and marine animals	Natural products of Brazilian biodiversity. Developed by the São Paulo State University and the University of São Paulo.	[42, 43]
SistematX (Brazil)	9514	Plants	Database composed of secondary metabolites and developed at the Federal University of Paraiba.	[44, 45]
UEFS (Brazil)	503	Plants	Natural products that have been published separately, but there is no common publication nor public database for it. Developed at the State University of Feira de Santana.	[46]
NPDBEjeCol (Colombia)	200	Plants Plants‐derived food	Natural products and foods derived from plants present in the Eje Cafetero Región of Colombia, database created and curated at the Technological University of Pereira.	[47]
NAPRORE‐CR (Costa Rica)	1161	Plants Microorganisms	Developed in the CBio3 and LaToxCIA Laboratories of the University of Costa Rica.	a
LAIPNUDELSAV (El Salvador)	214	Plants	Developed by the Research Laboratory in Natural Products of the University of El Salvador.	a
UNIIQUIM (Mexico)	1112	Plants	Natural products isolated and characterized at the Institute of Chemistry of the National Autonomous University of Mexico.	[48]
BIOFACQUIM (Mexico)	750	Plants Fungi Propolis Marine animals	Natural products isolated and characterized in Mexico at the School of Chemistry of the National Autonomous University of Mexico and other Mexican institutions.	[49, 50]
NAPROPMA (Panama)	531	Plants Terrestrial and marine animals Endophytic Fungi	Natural products that have been tested in over twenty‐five in vitro and in vivo bioassays for different therapeutic targets. Developed at the University of Panama.	[35, 36]
PeruNPDB (Peru)	280	Animals Plants	Natural products representative of Peruvian biodiversity. Created and curated at the Catholic University of Santa Maria.	[51]

a The database has not been published yet.

The UNPDB was chosen as reference NPs database since it is one of the largest open access NPs databases [23, 24]. At the end of the curation process, a total of 153,325 compounds were retained from the UNPDB. The approved drugs database version 5.1.12 (released by DrugBank in March 2024) [41] originally contains 2,769 drugs. After the curation process and elimination of the drugs classified as experimental, investigational, withdrawn, illicit, and nutraceutical the number of drugs was reduced to 924. The compounds that included in their classification the label ‘experimental’ were removed because this group not just contains molecules with a direct therapeutic application, but also includes excipients, additives, diagnostic agents, cosmetics, and dietary supplements. The drugs labeled as ‘investigational’ were removed since not all of them are approved by the FDA; nonetheless, they may be approved by other regulatory agencies. From the 924 drugs, 112 compounds (12.12%) include in its label ‘veterinary approved.’ After the curation process, the remaining molecules were small‐molecules, that is why this database is referred herein as ’approved small‐molecule drugs’.

### 3.2 ADMET Profile

**FIGURE 1:**
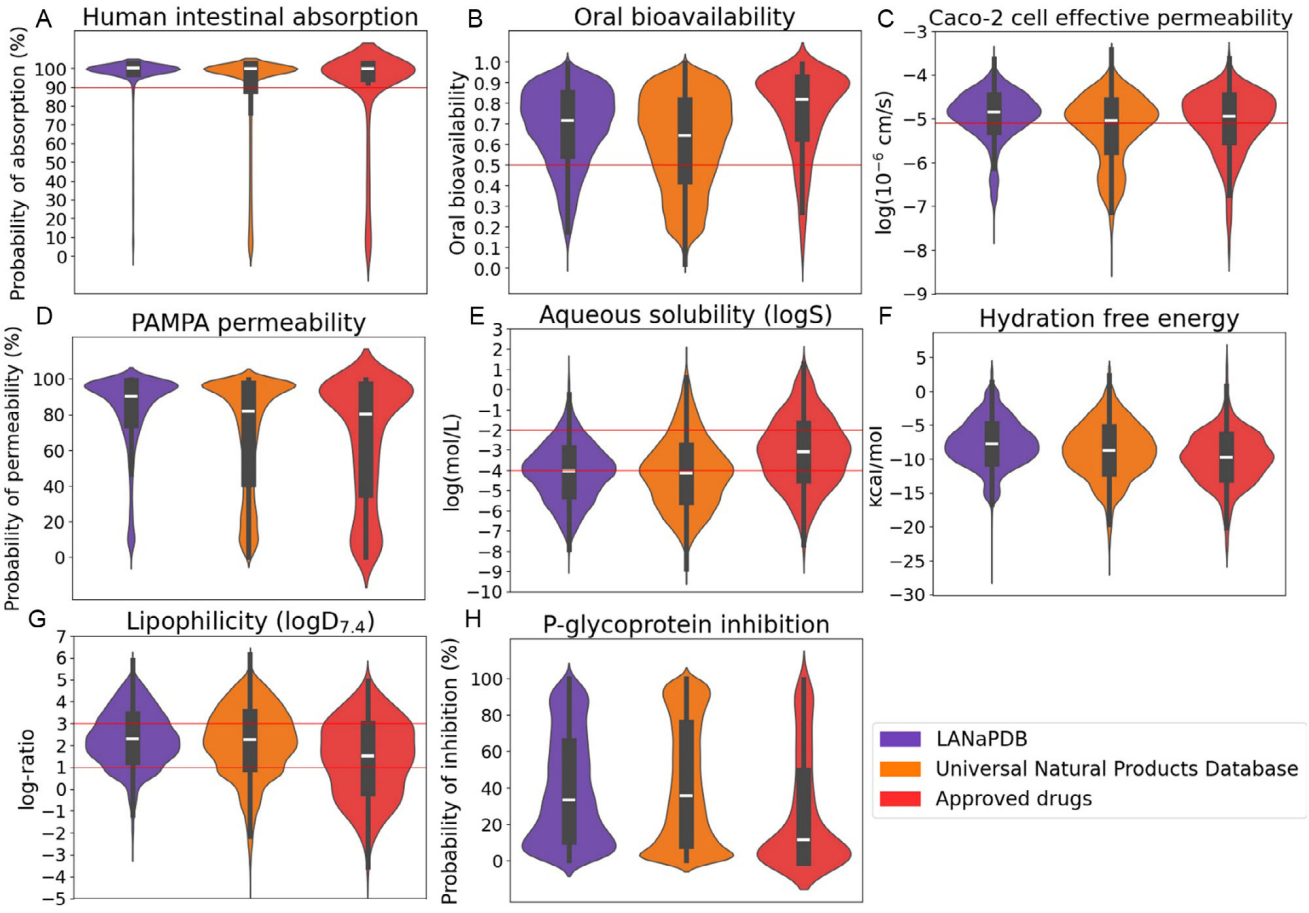
Violin and box plots of the predicted absorption parameters calculated with ADMET‐AI of LANaPDB, the Universal Natural Product database, and the approved small‐molecule drugs. Parallel artificial membrane permeability assay (PAMPA). Horizontal lines depict the limits between desirable and not desirable values of the following parameters: (A) human intestinal absorption [52], (B) oral bioavailability [53], (C) Caco‐2 cell effective permeability [54], (D) PAMPA permeability, (E) aqueous solubility (logS), (F) hydration free energy, [55, 56], and (G) lipophilicity (logD_7.4_) [57], (H) P‐glycoprotein inhibition.

**FIGURE 2:**
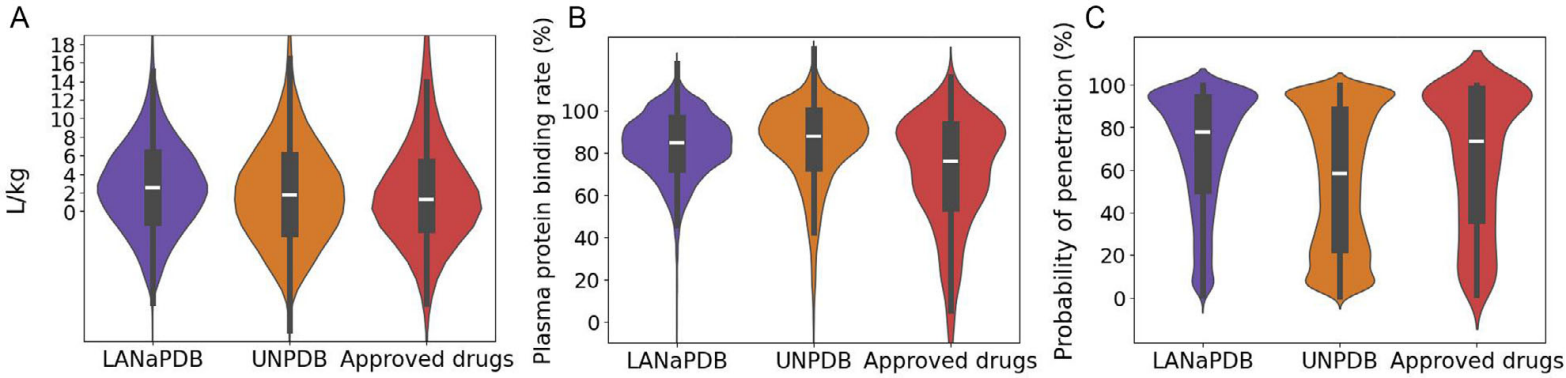
Violin and box plots of the predicted distribution parameters calculated with ADMET‐AI of LANaPDB, the Universal Natural Product database, and the approved small‐molecule drugs. (A) Volume of distribution at steady state; (B) plasma protein binding rate; and (C) blood−brain barrier penetration.

**FIGURE 3:**
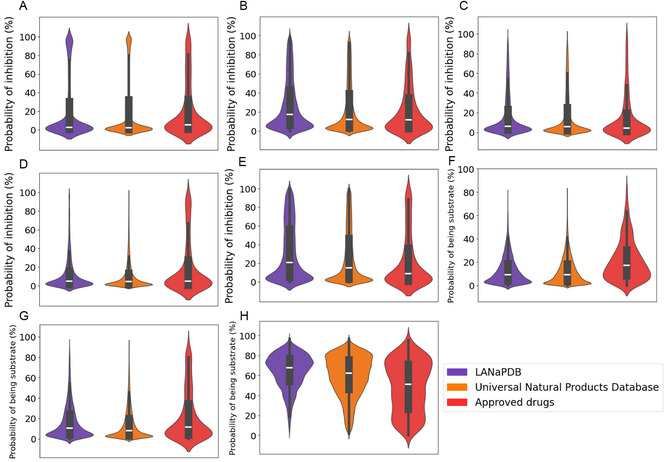
Violin and box plots of the predicted metabolism parameters calculated with ADMET‐AI of LANaPDB, the Universal Natural Product database, and the approved small‐molecule drugs. Cytochrome P450 (CYP). (A) CYP1A2 inhibition; (B) CYP2C19 inhibition; (C) CYP2C9 inhibition; (D) CYP2D6 inhibition; (E) CYP3A4 inhibition; (F) CYP2C9 substrate; (G) CYP2D6 substrate; and (H) CYP3A4 substrate.

**FIGURE 4:**
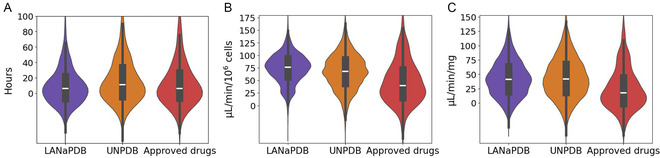
Violin and box plots of the predicted excretion parameters calculated with ADMET‐AI of LANaPDB, the Universal Natural Product database, and the approved small‐molecule drugs. (A) Half life (t_1/2_); (B) drug clearance (hepatocute); and (C) drug clearance (microsome).

**FIGURE 5:**
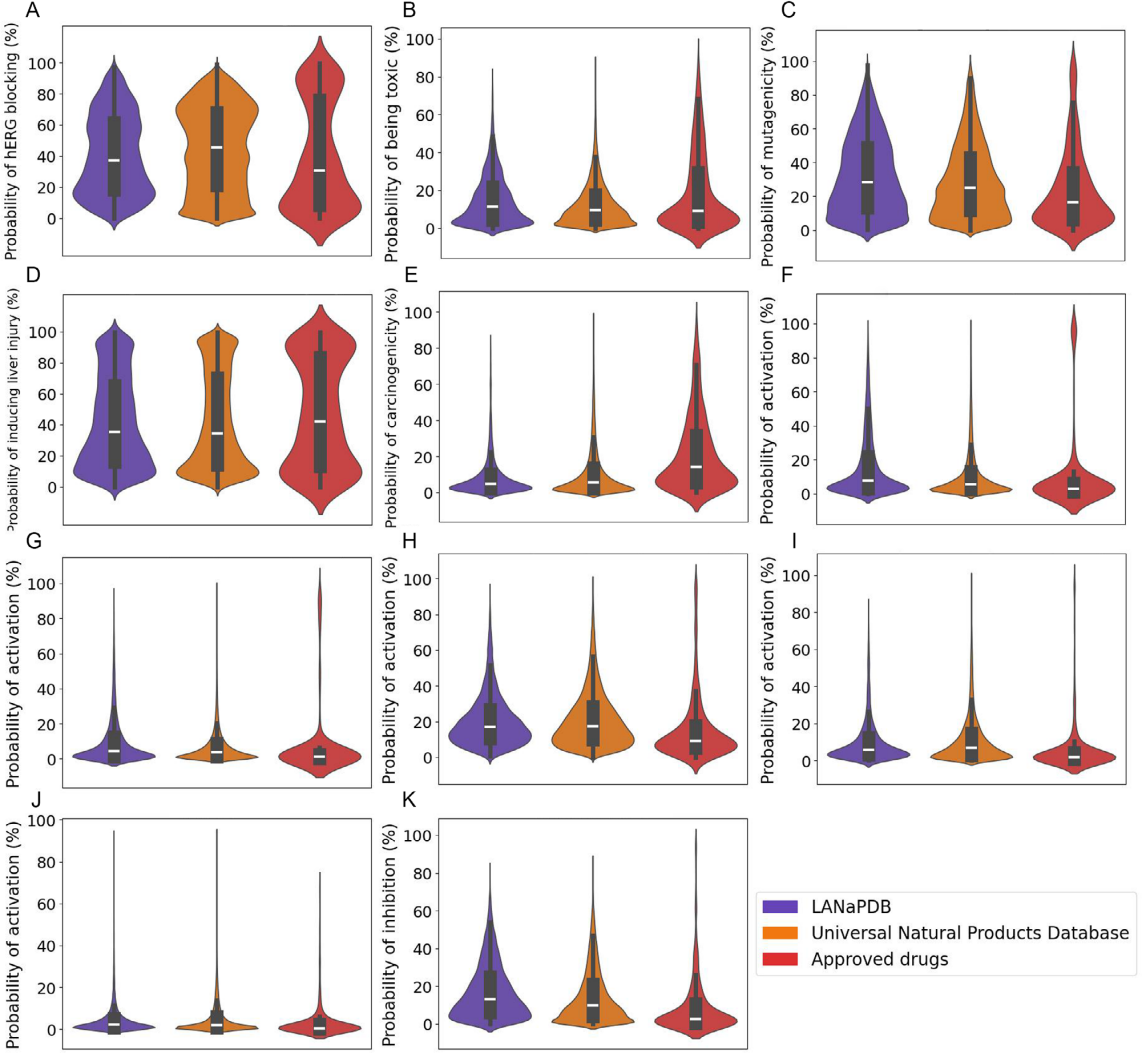
Violin and box plots of eleven predicted toxicity parameters calculated with ADMET‐AI of LANaPDB, the Universal Natural Product database, and the approved small‐molecule drugs. Human ether‐a‐go–go‐related gene (hERG), ligand binding domain (LBD), and peroxisome proliferator‐activated receptor gamma (PPAR‐*γ*). (A) hERG blocking; (B) clinical toxicity; (C) mutagenicity (Ames); (D) drug‐induced liver injury; (E) carcinogenicity; (F) androgen receptor (full‐length); (G) androgen receptor (LBD); (H) estrogen receptor *α* (full‐length); (I) estrogen receptor *α* (LBD); (J) PPAR‐*γ*; and (K) Aromatase.

**FIGURE 6:**
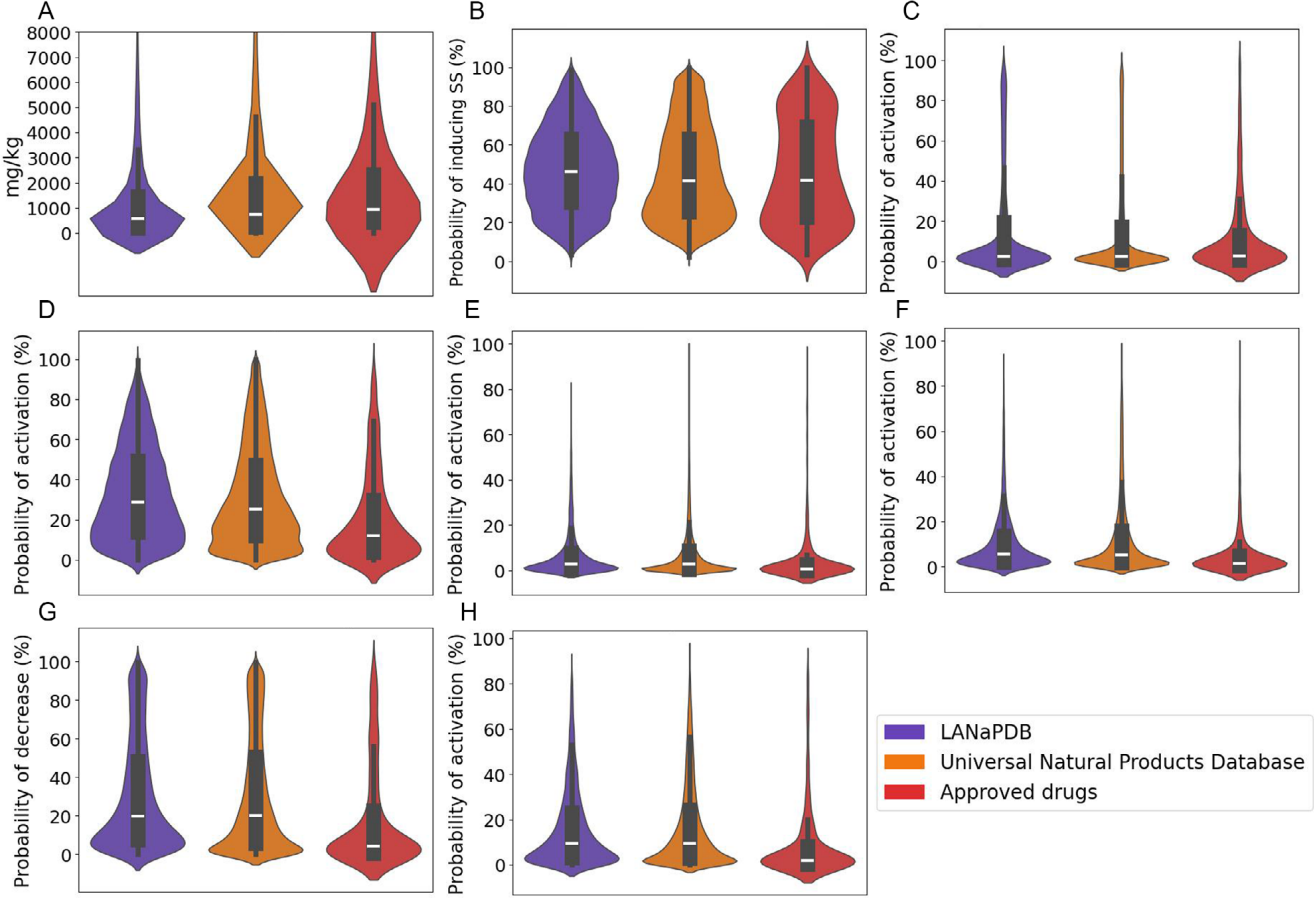
Violin and box plots of eight predicted toxicity parameters calculated with ADMET‐AI of LANaPDB, the Universal Natural Product database, and the approved small‐molecule drugs. ATPase family AAA domain‐containing protein 5 (ATAD5), median lethal dose (LD_50_) mitochondrial membrane potential (MMP), nuclear factor erythroid 2‐related factor 2 (Nrf2), and skin sensitization (SS). (A) Acute toxicity (LD_50_); (B) skin sensitization; (C) aryl hydrocarbon receptor; (D) Nrf2/Antioxidant responsive element; (E) ATAD5; (F) heat shock transcription factors; (G) mitochondrial membrane potential; and (H) p53.

Forty‐one ADMET‐related parameters were calculated with ADMET‐AI for LANaPDB, the UNPDB, and the approved small‐molecule drugs version 5.1.12 (released by DrugBank in March 2024). ADMET‐AI uses neural networks which were trained with experimental data for each one of the forty‐one ADMET‐related parameters [19]. The applicability domain of the ADMET‐AI neural networks is the small‐molecule organic compounds, since the training datasets were comprised of this class of compounds. Taking into consideration that ADMET‐AI was trained with organic small‐molecules, including approved drugs, and LANaPDB is composed almost totally by organic small‐molecules (0.56% of the compounds are aminoacids and peptides) [28, 29], it is considered valid to use ADMET‐AI to generate an *in silico* ADMET profile of LANaPDB. The obtained ADMET profile of the three databases is very similar (Figures 1, 2, 3, 4, 5–6). Previously, it was shown that LANaPDB is highly similar to the approved small‐molecule drugs considering either physicochemical properties and molecular structures [28, 29], and the similarity principle states that similar compounds have similar properties [58]. Taking into account the chemical space described by six physicochemical properties of pharmaceutical interest (lipophilicity, molecular weight (MW), polar surface area (PSA), number of rotatable bonds (Rb), hydrogen bond acceptors (HBA), and hydrogen bond donors (HBD)), it was discovered that LANaPDB totally overlaps with the approved drugs. Moreover, considering the chemical space described by three different fingerprints (MACCS keys (166‐bit), Morgan2 (2048‐bit), and MAP4), it was found that LANaPDB mostly overlaps with the small‐molecule approved drugs [28, 29]. Furthermore, it is very similar the molecular complexity of the small‐molecule approved drugs compared to LANaPDB [28] and the other reference datasets of bioactive organic small‐molecules [59]. The similarity of LANaPDB and the UNPDB to the small‐molecule approved drugs can also be attributed to that, up to 2019, 66.8% of all the approved drugs (without including biologicals and vaccines), not just the ones approved by the FDA, were NPs and NP derivatives [1].

The data distribution of the 41 ADMET parameters is represented with box plots and violin plots (Figure 1, 2, 3, 4, 5‐6). Violin plots were constructed to provide additional insights into the distribution of the data. The median value for every ADMET parameter is represented with a horizontal white line inside the interquartile range (IQR) of every box plot (Figures 1, 2, 3, 4, 5–6). The boxplot statistics, maximum value (Max), quartile 3 (Q_3_), median, quartile 1 (Q_1_), and minimum value (Min) are in the Supporting Information (Tables S1–S5).

In the prediction of some ADMET‐related parameters, compounds were found with values outside the expected range. These deviations can be attributed to limitations of the neural network models, including possible extrapolation errors or insufficient constraints during training. To address this, postprocessing corrections may be applied to the original neural networks. Nonetheless, it must be taken into account that each of the forty‐one ADMET‐related parameters is predicted by a different neural network; therefore, corrections should be applied only to the specific networks that produced outlier values. It is pointed out that the volume of distribution at steady state is the main ADMET‐related parameter with outlier values, and to a lesser degree the three distribution parameters. Therefore, it is advised to make the necessary adjustments or corrections to future versions of the neural networks that predict these parameters. The implausible predicted values were retained but are considered as outliers.

#### 3.2.1 Absorption

##### 3.2.1.1 Human Intestinal Absorption and Oral Bioavailability

Regarding all 41 ADMET‐related parameters, each one is predicted with neural networks trained with experimental data. The absorption is predicted with two different *in vivo* parameters (Figure 1A,B) and two *in vitro* parameters (Figure 1C,D). The *in vivo* parameters include the human intestinal absorption (Figure 1A) [60] and the oral bioavailability (Figure 1B) [61]. The human intestinal absorption was determined from compounds that are believed to be transported by passive diffusion [60]. The oral bioavailability experimentally is determined by measuring the fraction of the compound that reaches the systemic circulation after an oral administration [53]. It is defined by the FDA as the rate and extent to which the active ingredient or active moiety is absorbed from a drug product and becomes available at the site of drug action [62]. The oral bioavailability (Figure 1B) expands the information obtained in the previous parameter (Figure 1A), predicting not just if the compound is absorbed, but also determines the fraction of the compound that reaches the systemic circulation. The recommended values of the human intestinal absorption are above 90% [52], and for the oral bioavailability, there is no universal criterion to define high and/or low bioavailable compounds, nonetheless, above 0.5 or 50% has been considered for high oral bioavailability [53]. The results of the predicted human intestinal absorption (Figure 1A) and oral bioavailability indicate that most compounds in the three databases have their values above the 90% and 0.5 recommended thresholds (human intestinal absorption: LANaPDB 91.01%, UNPDB 75.21% and approved drugs 80.95% and oral bioavailability: LANaPDB 82.45%, UNPDB 68.87% and approved drugs 87.01%). Therefore, the results of both absorption parameters indicate that most compounds are orally absorbed in the three databases. The LANaPDB molecules with lower values of oral bioavailability can still be considered in the drug discovery process since there are strategies to improve the oral drug bioavailability such as prodrug strategies, lead optimization, and formulation design [63].

##### 3.2.1.2 Caco‐2 Cell and PAMPA Permeability

The *in vitro* absorption was assessed with cellular and noncellular parameters (Figure 1C,D). The cellular parameter corresponds to the effective cell permeability, assessed using a monolayer of Caco‐2 cells (Figure 1C) [64], and the noncellular parameter is the parallel artificial membrane permeability assay (PAMPA) (Figure 1D) [65]. For the cell effective permeability (Figure 1C) are employed the Caco‐2 cell lines, derived from colorectal adenocarcinoma cells which display many of the morphological and functional properties of the enterocytes [64]. PAMPA (Figure 1D) is widely used in the early stages of drug discovery to predict the passive diffusion of compounds across phospholipid membranes. PAMPA is cost‐effective, robust, and highly reproducible [66]. It is considered that compounds with high Caco‐2 permeability have values equal or higher than 8 × 10^−6^ cm/s, that in logarithmic scale (log_10_) equals to −5.097, compounds with lower values are considered with moderate‐poor permeability [54]. The results showed that the three databases have a considerable percentage of compounds with high Caco‐2 cell effective permeability, with values higher than −5.097 (LANaPDB 69.67%, UNPDB 52.84% and approved drugs 59.52%). Interestingly, the violin plots of both *in vitro* absorption parameters (Figure 1C‐D) for the three databases are very similar, including the zone where most compounds are accumulated, that is around −5 in the Caco‐2 assay (Figure 1C) and almost 100% of PAMPA permeability which indicates a high Caco‐2 permeability and almost 100% of permeability probability in PAMPA (Figure 1D). Therefore, the LANaPDB and UNPDB compounds have a similar predicted *in vitro* permeability to the approved drugs. Moreover, the results of the *in vitro* parameters are in accordance with the results of the *in vivo* parameters showing a high predicted permeability for the three datasets.

##### 3.2.1.3 Aqueous Solubility

Regarding the aqueous solubility (Figure 1E) [55], compounds can be classified according to the obtained solubility values (LogS). Molecules with 0 and higher solubility values are highly soluble, those in the range of 0 to −2 are soluble, those in the range of −2 to −4 are slightly soluble, and insoluble if less than −4 [55]. Table 2 shows that in the three databases, most of the compounds are slightly soluble or insoluble. It is highlighted that 41.02% of the approved drugs are slightly soluble and 31.71% insoluble. Regardless, it is important to take into consideration that all the predictions made, including the solubility, just consider the structure of the molecules and not the pharmaceutical formulation that is present in the approved drugs that can improve the solubility. Therefore, the approved drugs that are slightly soluble or insoluble were able to reach the market after undergoing solubility enhancement techniques related to the pharmaceutical formulation. Some of the techniques to improve the solubility include particle size reduction, crystal habit modification, drug dispersion in carriers, and solubilization by surfactants. Furthermore, in 2023 were extensively reviewed the different techniques to enhance the solubility [67]. The fact that LANaPDB and the UNPDB follow the same tendency of the approved drugs, having most of its compounds as slightly soluble and insoluble, can be attributed to the similarity of the approved drugs to the NPs. As previously mentioned, 66.8% of the worldwide small‐molecule approved drugs are NPs and NP derivatives [1], and the similarity principle states that similar compounds have similar properties [58].

**TABLE 2:** Percentage of compounds in LANaPDB and reference libraries within the solubility ranges.

Database	Highly soluble	Soluble	Slightly soluble	Insoluble
	0 or higher	0 to −2	−2 to −4	−4 or less
LANaPDB	0.37%	6.47%	42.33%	50.83%
UNPDB	1.47%	10.87%	34.60%	53.06%
Approved drugs	4.65%	22.62%	41.02%	31.71%

##### 3.2.1.4 Hydration Free Energy

Another parameter that was calculated and it is highly related to the aqueous solubility is the hydration free energy (Figure 1F) [68] which is defined as the change in free energy associated with transferring the solute of interest from a dilute vapor phase into water [69]. This parameter is a key indicator of a molecule's solvation behavior in aqueous environments. More negative hydration free energy values indicate stronger solute‐water interactions, which correlate with higher aqueous solubility. Less negative or even positive values indicate little or no aqueous solubility. In the three databases, the hydration‐free energy values of most compounds are concentrated around their respective median values (LANaPDB −7.81, UNPDB −8.70, and approved drugs −9.80) which is a zone where the compounds can be considered slightly soluble. Therefore, the obtained results of hydration‐free (Figure 1F) energy and aqueous solubility (Figure 1E, Table 2) are in accordance, indicating that most of the compounds in the three databases are poorly soluble.

##### 3.2.1.5 Lipophilicity (LogD_7.4_)

The lipophilicity (LogD_7.4_) [70] is another parameter that was predicted (Figure 1G) which takes into account not just the neutral compound as the logP does, but also considers the concentration of the ionized species at the physiological pH (7.4) [71]. Table 3 shows that the three databases have their compounds concentrated around one to three, a range that is expected to correspond to a favorable pharmacokinetic profile [57]. The percentage of compounds inside this range is the following: LANaPDB 54.31%, UNPDB 45.20%, and approved drugs 40.37%. Therefore, LANaPDB is the database with the highest proportion of compounds inside the optimal range of one to three. Interestingly, the small‐molecule approved drugs are the dataset with the highest proportion of compounds (59.63%) outside the optimal range of one to three. The fact that most of the approved drugs are outside the optimal range one to three, is that the LogD_7.4_ does not take into account the pharmaceutical formulation that can address the problems related to a nonoptimal lipophilicity. There are formulation strategies focused on the pharmacokinetic profile improvement [72], including the enhancement of the intestinal absorption of highly lipophilic compounds [73]. Moreover, there is an alternative route for delivering highly lipophilic drugs (logP > 5) to the body circulation, the intestinal lymphatic transport which avoids the hepatic first‐pass metabolism, therefore, increasing the oral bioavailability [73]. Besides, for highly lipophilic molecules can be considered alternative drug delivery routes, such as the nasal route, where lipophilic molecules are generally satisfactorily transported through the nasal mucosa, via the transcellular pathway including receptor, carrier, and vesicle‐mediated routes [73]. Another alternative is the intramuscular delivery route because the lipophilicity of a drug favors rapid diffusion into the capillaries, nonetheless, the drug must retain a degree of water solubility at physiologic pH to prevent precipitation at the injection site [74]. Regardless, there are other mechanisms of absorption that improve the bioavailability of compounds with poorly cellular passive permeability, such as the active [75] and paracellular transport [76, 77]. The paracellular pathway involves the intercellular junctions and has two mechanisms, the epithelial leak pathway, which is size‐dependent but not charge‐dependent, and allows the absorption of large compounds such as lipopolysaccharides and proteins. The second mechanism is the pore pathway, which is size‐ and charge‐dependent, it allows the permeation of polar drugs and excludes molecules larger than 4 Å. Regarding the charge selectivity are preferred the cationic over anionic drugs [73]. Amphoteric drugs such as acyclovir, amdinocillin, ganciclovir, piroxicam, and trovafloxacin, undergo extensive paracellular transport [78]. Therefore, the LANaPDB compounds with poor solubility and high lipophilicity still can be considered as candidates for the drug design process, additionally, taking into account that many of the approved drugs fall into this category (Tables 2 and 3).

**TABLE 3:** Percentage of compounds in LANaPDB and reference libraries within the lipophilicity (LogD_7.4_) ranges.

Database	Lipophilicity (LogD_7.4_)		
	1 or less	1 to 3	3 or high
LANaPDB	14.90%	54.31%	30.79%
UNPDB	22.72%	45.20%	32.08%
Approved drugs	40.26%	40.37%	19.37%

##### 3.2.1.6 P‐Glycoprotein Inhibition

P‐glycoprotein (P‐gp) inhibition is the last absorption parameter that was determined (Figure 1H). P‐gp is an efflux pump responsible for pumping out substances of the cell, including small‐molecule drugs. In the intestinal tract, it is mainly expressed in the ileum and colon which is the main cause for the reduced drug absorption for a range of molecules, such as cyclosporin, tacrolimus, and paclitaxel [73]. P‐gp inhibition can improve the drug oral bioavailability. The three databases contain compounds predicted as either inhibitors or not inhibitors. Besides, LANaPDB and the UNPDB have a major proportion of compounds predicted as inhibitors compared to the approved drugs. Moreover, in the three databases, the compounds are concentrated mainly in a noninhibitor zone. To date, the inhibition of P‐gp is a promising strategy against multi‐drug resistance in cancer therapy, therefore, the compounds predicted as P‐gp inhibitors can be considered as candidates in the design of novel compounds against this target [79].

#### 3.2.2 Distribution

##### 3.2.2.1 Volume of Distribution at Steady State

Three parameters were determined to predict the distribution of the LANaPDB compounds, volume of distribution at steady state (VDss) (Figure 2A) [80], plasma protein binding rate (PPBR) (Figure 2B) [81], and blood−brain barrier (BBB) penetration (Figure 2C) [81]. The VDss is the total amount of drug in the body divided by its plasmatic concentration at equilibrium. The VDss represents an individual drug's propensity to either remain in the plasma or redistribute to other tissue compartments. VDss values higher than one indicate a high propensity to leave the plasma and enter other tissues. Therefore, a higher dose of a drug is required to achieve a given plasma concentration. VDss values lower than one suggest a high propensity to remain in the plasma. Consequently, a lower dose of a drug is required to achieve a given plasma concentration. The role of the VDss, together with clearance (Figure 4B,C), determines the frequency of dosing. A compound with a lower VDss may have to be dosed more frequently (at a lower dose) than a compound with a larger VDss, which would require to be dosed less frequently. There is no guarantee that a compound will reach a site of action and be pharmacologically active or not, on the basis of its VDss. Therefore, high values of VDss do not mean ‘good’ and low values ‘poor’ or ‘problematic efficacy.’ Therefore, there is not a certain threshold value of VDss that indicates that a compound is ‘good’ or ‘bad,’ as previously said, VDss is used to determine the frequency of dosing [80, 82]. The distribution of the VDss values is very similar in the three datasets, being the violin plots of LANaPDB and the UNPDB very similar to the approved drugs. Besides, a high proportion of the compounds in LANaPDB (63.57%), UNPDB (55.33%), and approved drugs (53.57%) have VDss values higher than one, indicating a major propensity of the compounds to remain in tissue compartments rather than in plasma. Therefore, most of the LANaPDB compounds would require a higher dose to achieve a given plasma concentration. Nonetheless, the major propensity to remain in tissue compartments rather than in plasma can facilitate the compounds’ ability to reach their therapeutic targets, as long as these are not located in plasma or in organs with limited accessibility.

##### 3.2.2.2 PPBR

The PPBR was determined for the three databases (Figure 2B). Small‐molecule drugs can bind to plasma proteins, and just the unbound fraction has the capacity to reach the desired site of action. The main binding protein in plasma is albumin, the next most abundant is the *α*_1_‐ acid glycoprotein, and in a minor proportion the *α*‐, *β*‐, *γ*‐globulins, and the lipoproteins [83]. Acidic drugs mainly bind to albumin, and basic lipophilic drugs mainly bind to *α*_1_‐acid glycoprotein and lipoproteins [84]. The predicted PPBR considers the union to all the plasmatic proteins. The results indicated that most of the compounds in LANaPDB (80.94%), the UNPDB (81.08%), and approved drugs (57.14%) have a high probability (70% or higher) of binding to plasmatic proteins. Besides, the approved drugs (13.74%) present a higher proportion of compounds with lower PPBR (40% or less) compared to LANaPDB (0.91%) and the UNPDB (3.90%). Regardless, some authors recommend that PPB should not be optimized through structural modifications or be used to develop structure–activity relationships (SAR), because this parameter has little effect on free drug concentration *in vivo* or efficacy in the clinic. The same authors claim that a highly bound compound to plasma proteins does not mean ‘bad,’ conversely, a highly free compound does not necessarily mean ‘good’ [85]. Nevertheless, it is important to consider that just the unbound fraction of the drug is capable of reaching the site of action, and the other fraction bound to plasmatic proteins cannot exert its therapeutic activity.

##### 3.2.2.3 BBB Penetration

The BBB penetration (Figure 2C) is the third ADMET‐related distribution parameter predicted. The BBB is a membrane that protects the central nervous system (CNS) by restricting the passage of solutes. This parameter must be taken into account in the drug design process either to ensure drug penetration into the CNS when targeting its components or to prevent it, thereby avoiding adverse drug reactions caused by CNS interactions [81]. The violin plots show in the three databases a concentration of the compounds near to 100% BBB probability of penetration. Besides, there is a high proportion of compounds in the three databases with a BBB probability of penetration higher than 70%, LANaPDB (60.27%), the UNPDB (41.03%), and approved drugs (53.14%). The proportion of compounds with 40% or lower BBB probability of penetration is less, LANaPDB (17.01%), the UNPDB (37.33%), and approved drugs (25.65%). Regardless, the lipophilicity of a compound can be modified to improve or decrease the BBB penetration. The lipid soluble drugs with MW among 400–600 Da, are able to cross the BBB. Nevertheless, it is important to take into account that high lipophilic compounds have a longer retention and duration of action in nontarget peripheral organs, causing considerable side effects. Therefore, another alternative to cross the BBB without increasing the lipophilicity is the use of specific drug delivery systems [86]. Consequently, even the LANaPDB compounds with low BBB probability of penetration can be considered to target the CNS. Nonetheless, it should be considered a potential risk of adverse events related to CNS interactions for those LANaPDB compounds with high BBB probability of penetration. It is highlighted that the P‐gp inhibition is a parameter that influences the BBB penetration, because this protein is expressed at the BBB [87]. Therefore, in the polypharmacy context, it should be considered the P‐gp inhibition which can lead to the penetration of compounds to the CNS whose entrance was hindered previously by P‐gp.

#### 3.2.3 Metabolism

The cytochrome P450 (CYP) family comprises 57 isozymes in humans. They metabolize approximately two‐thirds of known drugs in humans, with 80% of this attributable to five isozymes, 1A2, 2C19, 2C9, 2D6, and 3A4 [88]. CYP3A4 is the most abundant CYP3A enzyme in the liver and is responsible for the metabolism of approximately 30–50% of clinically used drugs [89]. These five isozymes are the predominant forms expressed in the liver responsible for first‐pass metabolism. Nonetheless, CYP enzymes are also in the intestines, kidneys, lungs, and brain [90]. CYP enzymes are responsible for oxidative transformation of endogenous and exogenous molecules [91]. The CYP enzymes transform the xenobiotics to facilitate the excretion through metabolism and may be the main route of elimination of many drugs. Nevertheless, most drugs undergo deactivation after CYP enzymes biotransformation. Moreover, in some cases, the CYP biotransformation can make a xenobiotic toxic or more toxic. Furthermore, CYP enzymes are responsible for the bioactivation of some prodrugs [92]. In the context of polypharmacy it is relevant to know if a drug inhibits the CYP enzymes, because this can affect the therapeutic activity of the drugs that undergo CYP metabolism. For instance, the inhibition of a CYP enzyme that bioactivates a prodrug will lead to exhibit little to no signs of benefit from the treatment. Furthermore, if a drug acts as a CYP inhibitor, then that causes other drugs to accumulate to toxic levels where overdoses and side effects may occur [92]. In the drug design process, knowing if a drug candidate is substrate of CYP enzymes may help in the design of prodrugs or to predict the metabolites of a certain xenobiotic. CYP enzymes display an inherent affinity for lipophilic substrates due to their lipophilic nature. Whereas, depending on the ionization states, lipophilic compounds also show inhibition potential against CYP enzymes [93].

Figure 3 shows the results of prediction of the interaction of the three dataset compounds against the main CYP enzymes. The results indicated that in the three databases, most compounds have a low probability to inhibit the CYP isozymes 1A2, 2C19, 2C9, 2D6, and 3A4 (Figure 3A–E). This can be appreciated in the violin plots (Figure 3A–E), where the compounds in the three databases are mainly distributed below 20% of probability of CYP inhibition. Interestingly, the violin plots of the three databases are similar, showing again the high similarity between the NPs and the small‐molecule approved drugs. As previously said, the inhibition of the CYP isozymes can lead a xenobiotic to its accumulation to toxic levels where overdoses and side effects may occur [92]. Therefore, in the context of polypharmacy, it is desirable that most of the LANaPDB compounds have low inhibition against the five CYP isozymes, if they would be considered as candidates in the design of new drugs.

In the case of the probability of being biotransformed by the CYP isozymes, most of the compounds of the three databases have a low probability (20% or low) of being substrate of the CYP2C9 and 2D6 (Figure 3E‐F). Conversely, the compounds of the three databases presented high probabilities of being substrates of CYP3A4 (Figure 3H). LANaPDB is the database with the highest proportion of compounds (65.43%) with a probability of 70% or higher of being substrate of CYP3A4, followed by the UNPDB (53.92%) and the approved drugs (40.91%). The higher percentage of CYP3A4 substrates, compared to the small number of CYP2C9 and 2D6 substrates, can be attributed to the structural differences between these cytochromes. CYP3A4 belongs to a different CYP family (family three) than CYP2C9 and 2D6 (family two), therefore, the identity of the amino acid primary chain is 40% or lower among both families [94]. Besides, there is large geometric variation at the active site between CYP3A4 and CYP2C9 [95]. The approved drugs, compared to the other two datasets, presented a violin plot (Figure 3H) with the compounds more equally distributed among high and low probabilities of CYP3A4 inhibition. The above can be attributed to the higher structural diversity of the small‐molecule approved drugs compared to the NPs, including LANaPDB [28].

#### 3.2.4 Excretion

##### 3.2.4.1 Half‐Life

Half‐life is the time required for the concentration of a drug (typically in blood or plasma) to reduce to half of its initial value when the concentrations of the drug are in simple exponential (log−linear) decline. A half‐life of 12−48 h is generally ideal for once daily dosing of oral drugs. If the half‐life is too short, it may require more frequent dosing in order to maintain desired exposures and avoid unnecessarily high peak concentrations. Conversely, if the half‐life is too long, the time over which accumulation and subsequent elimination occur may be prolonged. This may pose problems with managing adverse drug reactions, and it is also difficult to run crossover clinical studies when the drug half‐life is long [96]. Figure 4A shows the results of the predicted half‐life, and Table 4 shows the percentage of compounds inside the recommended half‐life values. In the three datasets, approximately 30% of the compounds are inside the desirable half‐life range of 12−48 h. Moreover, in the three databases, approximately 10% of the compounds have a half‐life longer than 48 h. Furthermore, most compounds in the three databases have a half‐life lower than 12 h. That most approved drugs are below the recommended 12‐h half‐life can be attributed to the fact that the prediction just takes into account the chemical structure of the molecules, and the approved drugs have a pharmaceutical formulation that can address pharmacokinetic deficiencies such as a short half‐life. Actually, extended‐release pharmaceutical formulations are used to address the problem of short half‐life by extending its duration [97]. Nonetheless, there are other strategies to extend the half‐life, based on making structural modifications to molecules [98]. Therefore, the LANaPDB compounds with a half‐life lower than twelve hours are still worth to be considered as drug candidates with an once daily oral regimen. Regardless, there are currently commercialized drugs with experimentally reported very short (less than 1 h) and long half‐life (greater than 48 h, even up to 2 weeks) [99].

**TABLE 4:** Percentage of compounds in LANaPDB and reference libraries within half‐life ranges.

Database	Half‐life (hours)		
	12 or less	12 to 48	48 or high
LANaPDB	60.89%	31.50%	7.61%
UNPDB	51.35%	33.96%	14.69%
Approved drugs	60.06%	28.25%	11.49%

##### 3.2.4.2 Drug Clearance

Clearance quantifies the irreversible removal of a drug from the measured matrix (typically blood or plasma) [100]. Liver microsomes and hepatocytes are commonly used in *in vitro* models for clearance prediction. Liver microsomes are rich in metabolizing enzymes, especially CYP450, but with the limitation of incomplete metabolic pathways compared to the hepatocytes. Nonetheless, they are extensively used for their low cost compared to hepatocytes. Hepatocytes have more metabolic enzymes and cofactors for the different clearance pathways. Besides CYP450, hepatocytes contain several enzymes of Phase I and Phase II, such as aldehyde oxidase, monoamine oxidase, and uridine 5′‐diphospho‐glucuronosyltransferase. Additionally, hepatocytes maintain the cell membrane structure and retain most transporter functions, providing a closer approximation to the *in vivo* system compared to liver microsomes [101]. Figure 4 shows the results of the predicted hepatocyte (Figure 4B) and microsomal clearance (Figure 4C). The hepatocyte and microsomal clearance of both NP databases are higher than the approved drugs. Therefore, the NPs are more susceptible to hepatic metabolism than the approved drugs which is expected considering that most of the small‐molecule approved drugs have been gone through an optimization process [1], improving parameters of their pharmacokinetic profile, including the metabolic stability against the hepatic enzymes. Furthermore, the results indicate that LANaPDB and the UNPDB undergo Phase I and Phase II metabolism which is in accordance with the prediction that most compounds in the two NP databases are CYP3A4 substrates (Phase I metabolism) (Figure 3H).

#### 3.2.5 Toxicity

##### 3.2.5.1 hERG Blocking

Nineteen toxicity ADMET‐related parameters were predicted for the three databases (Figures 5 and 6). The first parameter is the blockade of human ether‐à‐go–go related gene (hERG) channel (Figure 5A). The hERG potassium channel is located in the heart and plays a major role in the regulation of the exchange of cardiac action potential and resting potential. Its blockade is related to cardiac arrhythmias, therefore, several drugs which block this channel have been withdrawn or severely restricted in availability [102]. LANaPDB and approved drugs have the biggest concentration of compounds below 20% of probability of hERG channel blocking. Interestingly, there is another major concentration of compounds in the approved drugs with a blocking probability above 80%. Among these compounds are the actual commercialized drugs flunarizine [103], pimozide [104], and perphenazine [105] which are potent hERG channel blockers. The cavity of the hERG channel is large and promiscuous, therefore, a large number of bioactive molecules have a certain probability to bind to this channel. Regardless, there are successful optimizations reported in the literature to decrease the affinity of a compound to the hERG channel, among them decreasing lipophilicity, lowering basicity, increasing rigidity, alteration of *π*‐*π* interactions, among others [105]. Thus, the LANaPDB compounds that are predicted as hERG channel inhibitors still can be considered as drug candidates, because the affinity can be reduced through an optimization process.

##### 3.2.5.2 Clinical Toxicity

Another predicted parameter is the clinical toxicity (Figure 5B) which neural network was trained with compounds that failed clinical trials due to its toxicity and also with drugs that are associated with successful trials [106]. The results indicated that most of the LANaPDB (79.61%) and the UNPDB (86.92%) compounds have a probability less than 25% of being toxic.

##### 3.2.5.3 Mutagenicity

The mutagenicity (Ames) is another predicted parameter (Figure 5C). The Ames test is the most widely used assay to determine the mutagenicity. This neural network was trained with experimental data coming from the Ames assay [107]. The violin plots (Figure 5C) show that the compounds in the three databases are distributed below a probability of 30% of mutagenicity, LANaPDB (52.31%), UNPDB (58.43%), and small‐molecule approved drugs (70.02%). Regardless, some toxicophore substructures associated with mutagenicity in the Ames test have been identified, and the avoidance of these substructures can lead to decrease the probability of mutagenicity [108].

##### 3.2.5.4 Drug‐Induced Liver Injury (DILI)

DILI (Figure 5D) has been reported as the main cause of compound failure in Phase II drug development and postmarket drug withdrawals, label changes, and use restrictions [109]. In 2024, a review article was published that highlights the various drugs that cause hepatotoxicity, their mechanisms of liver injury, and effects [110]. The knowledge from the above review can be applied during the drug discovery process to reduce the probability of DILI. The results of the DILI prediction (Figure 5D) show that the compounds in the three databases are mainly distributed below 30% of DILI probability. Furthermore, the approved drugs present another major concentration of compounds above 70% of DILI probability, which is attributed to the actually commercialized drugs with reported hepatotoxic potential [110].

##### 3.2.5.5 Carcinogenicity

Carcinogenicity is defined as the ability or tendency of a chemical to generate tumors, whether benign or malignant, increase their malignancy or accelerate tumor occurrence [111]. The prediction of carcinogenicity (Figure 5E) indicates that most of the LANaPDB (88.75%), the UNPDB (83.19%), and small‐molecule approved drugs (60.17%) compounds are below 20% of carcinogenicity probability. It is highlighted that the small‐molecule approved drugs have a major proportion of compounds with probability above 20% of carcinogenicity compared with the two NP databases. There are currently commercialized drugs with carcinogenic potential, including analgesics, antipyretics, immunomodulators, even anticancer agents [112].

##### 3.2.5.6 Androgen and Estrogen Receptors, PPAR‐*γ*, and Aromatase

Six toxicity parameters related to reproductive dysfunction were calculated which are the probability of activation of the following three receptors, androgen receptor (AR) full‐length (Figure 5F) and ligand binding domain (LBD) (Figure 5G), estrogen receptor alpha (ER*α*) full‐length (Figure 5H) and LBD (Figure 5I), peroxisome proliferator‐activated receptor gamma (PPAR‐*γ*) (Figure 5J), and the inhibition of the aromatase enzyme (Figure 5K). For the AR and ER*α,* the respective four neural networks were trained with data coming from *in vitro* assays that use the LBD and the full‐length receptors, the latter being more representative of the physiological conditions. The information regarding both the LBD and full‐length receptor may be helpful to identify if a molecule acts at the ligand‐binding site or allosterically. The three receptors and the enzyme are expressed in females and males, but the level of expression is variable. Some hormones act at the AR [113, 114, 115]**,** ER*α* [115], PPAR‐*γ* [116, 117], and aromatase [118, 119], playing a key role in the normal development of the reproductive organs, and a disruption can lead to reproductive dysfunctions such as infertility or interfering in the normal development of the reproductive organs in females and males. The results indicate that for the three receptors (Figure 5F,J), the probability of activation and inhibition for the aromatase enzyme (Figure 5K) is less than 20% for most compounds in the three datasets. Therefore, it can be considered that the compounds in the three databases have a predicted low probability of causing reproductive dysfunctions or interfering in the normal development of the reproductive organs through the AR, ER*α*, PPAR‐*γ,* and the aromatase.

##### 3.2.5.7 Acute Toxicity (LD_50_)

Acute oral toxicity is generally associated with a single exposure and occurs within a relatively short period of time, typically less than 24 h. It is quantified with the median lethal dose (LD_50_) which refers to the single oral dose that is predicted to cause death in 50% of the test animals [120]. The units of the LD_50_ log(1/(mol/kg)) were transformed to mg/kg (Figure 5F) to allow the classification of the compounds according to the United States Environmental Protection Agency (US EPA) (Table 5). The first category corresponds to highly toxicity, second to moderate toxicity, third to slightly toxicity, and the fourth to practically nontoxicity [121]. In the three databases most compounds are in the third category associated with slightly toxicity, the following most populated category is the second associated with moderate toxicity. Therefore, in the three databases, most compounds are moderate and slightly toxic. Regardless, the NPs that are in the fourth and third category should be prioritized as drug candidates over the compounds of the second and first category.

**TABLE 5:** Percentage of compounds in LANaPDB and reference libraries within the US EPA ranges.

Database	Toxicity category (US EPA)			
	I	II	III	IV
	50 or less	50 to 500	500 to 5000	5000 or higher
LANaPDB	5.33%	41.89%	46.83%	5.95%
UNPDB	5.90%	34.83%	49.70%	9.56%
Approved drugs	1.73%	28.68%	58.77%	10.82%

##### 3.2.5.8 Skin Sensitization

Many chemicals cause their adverse effects through skin contact, repetitive exposure to a chemical agent can induce an immune reaction that leads to skin sensitization. The mechanism of skin sensitization involves haptens that can covalently bind to cutaneous carrier proteins to form immunogenic hapten−protein complexes. Haptens are known to bear lipophilic moieties and have low MW (usually lower than 500 Da), allowing them to easily cross the first line of defense of the skin, the stratum corneum barrier [122]. They also have an electrophilic moiety that allows them to form an immunogenic complex with proteins. Furthermore, pre‐haptens (require abiotic activation such as contact with air oxygen or radiation) and pro‐haptens (require biotic modifications of specific cutaneous enzymes) which are nonelectrophilic can be converted into electrophilic haptens [123]. Figure 6B shows the results of the predicted skin sensitization. Half of the compounds in the UNPDB (48.07%) and the small‐molecule approved drugs (48.59%) are below 40% of probability of inducing skin sensitization. Moreover, the distribution of the LANaPDB compounds is wider, and most of the LANaPDB compounds (60.27%) are between 20% and 60% of skin sensitization probability. Approximately, one‐third (29.01%) of the LANaPDB compounds have a probability higher than 60% of inducing skin sensitization. Therefore, these results should be considered for the LANaPDB compounds that are intended to be developed as topical pharmaceutical formulations or cosmetics, avoiding the use of compounds for these formulations with highly predicted skin sensitization which can include pre‐haptens, pro‐haptens, and haptens. Moreover, the compounds with electrophilic moieties in general should be avoided for topical pharmaceutical formulations and cosmetics because of their potential of being haptens.

##### 3.2.5.9 Aryl Hydrocarbon Receptor (AHR)

The AHR is a ligand‐activated transcription factor originally identified as the target mediating the toxic effects of environmental pollutants including polycyclic aromatic hydrocarbons, polychlorinated biphenyls, and dioxins [124]. Its activation has been linked to adverse effects on cardiovascular diseases, hypertension, diabetes, obesity, kidney disease, and nonalcoholic fatty liver disease [125]. For years, AHR activation had been avoided in drug design, nonetheless, later was identified as a key regulator of the innate and adaptive immunity [124]. Consequently, in 2022 was approved the first AHR activator (tapinarof), to treat plaque psoriasis, an immune‐mediated disease [126]. Nowadays, it is highlighted the focus on the AHR as therapeutic target to treat multiple sclerosis, autoimmune encephalomyelitis, inflammatory bowel disease, asthma, and chronic obstructive pulmonary disease, some infectious diseases, and certain types of cancer [124]. The results show that LANaPDB (75.32%), the UNPDB (76.52%), and approved drugs (81.82%) have most of their compounds with a probability below 20% of ARE activation. Therefore, most of the LANaPDB compounds have a low probability of having adverse effects mediated by the AHR. Regardless, the few compounds that were predicted as AHR activators can be considered as candidates to treat the above‐mentioned diseases mediated by the AHR.

##### 3.2.5.10 Nrf2/Antioxidant Response Element

The nuclear factor erythroid 2‐related factor 2 (Nrf2) is the transcriptional master regulator of cellular responses against oxidative stress. Nrf2 regulates the expression of a multitude of antioxidant and Phase II enzyme genes. Therefore, the Nrf2 activation allows the identification of chemicals that stimulate oxidative stress. The compounds with predicted high probability of Nrf2 activation (Figure 6D) should be considered as chemicals that may stimulate oxidative stress. Chronic oxidative stress and the resultant oxidative damage have been implicated in many human diseases including cardiovascular disease, neurodegenerative diseases, diabetes, cancer, and the aging process [127]. The results (Figure 6D) indicate that most compounds in the three databases have a probability of activating Nrf2 of 40% or less, LANaPDB (64.65%), UNPDB (68.46%), and the small‐molecule approved drugs (81.17%). The oxidative stress is mainly driven by the formation and prolonged exposure to reactive oxygen species (ROS). Hence, for the LANaPDB compounds pretended to be developed as drug candidates, but predicted as chemicals that can stimulate the oxidative stress (high Nrf2 activation probability), can be considered the co‐administration of ROS inhibitors [128].

##### 3.2.5.11 ATAD5

ATPase Family AAA Domain‐Containing Protein 5 (ATAD5) is a biomarker for identifying genotoxic compounds (compounds with the ability of damaging the genetic information) [129], ATAD5 protein levels increase post transcriptionally in response to DNA damage [130]. The mechanism that involves ATAD5 in the promotion of genomic stability is complex and involves the interaction of ATAD5 with other proteins [131]. The results (Figure 6E) showed that most of the compounds in LANaPDB (79.69%), UNPDB (77.54%), and the small‐molecule approved drugs (88.53%) have a predicted probability less than 10% of activating the ATAD5 response. Although most of the LANaPDB compounds are predicted as nongenotoxic, according to the ATAD5 response, there are measures that can be taken to avoid genotoxicity. The ability to form covalent adducts with DNA is a characteristic of genotoxic compounds, and the chemical moieties with such capacity have been identified. There have been identified 57 structural alerts associated with covalent DNA binding and their mechanism of DNA adduct formation [132], and avoiding them can lead to preventing genotoxicity.

##### 3.2.5.12 Heat Shock Transcription Factors (HSFs)

The HSFs are direct transcriptional activators of genes regulated by thermal stress, encoding heat shock proteins [133]. HSFs bind to DNA cis‐regulatory motifs known as heat shock elements to mediate the transcriptional response of HSFs target genes [134]. Nonetheless, the HSFs response is not just activated by thermal stress, it is also an indicator of acute cell stress caused by extreme proteotoxic (condition or agent that causes damage to cellular proteins) insults such as oxidative stress, heavy metals, toxins, and bacterial infections. Therefore, the HSFs response has the function of protecting cells against proteotoxic stress [135]. The results (Figure 6F) indicate that the three databases have most of their compounds below 20% of HSFs activation, LANaPDB (84.73%), UNPDB (79.93%), and the small‐molecule approved drugs (93.61%). Consequently, the LANaPDB compounds have a low predicted probability of causing proteotoxic stress. Regardless, the induction of apoptosis by proteotoxic stress by small‐molecule drugs is suggested as an option to treat aggressive cancers with few therapeutic options, such as leiomyosarcoma [136].

##### 3.2.5.13 Mitochondrial Membrane Potential (MMP)

Drug‐induced mitochondrial dysfunction may result from increased production of ROS, altered mitochondrial permeability transition, impaired mitochondrial respiration, mitochondrial DNA damage, or inhibition of fatty acid *β*‐oxidation. The clinical manifestation depends on the specific drug and its effect on mitochondria. Given the ubiquitous presence of mitochondria and their central role in cellular metabolism, drug mitochondrial interactions may manifest clinically as hepatotoxicity, enteropathy, myelosuppression, lipodystrophy syndrome, or neuropsychiatric adverse effects, to name a few [137]. The decrease in the MMP is an indicator of mitochondrial impairment [138].

The results show that half of the LANaPDB (50.14%) and UNPDB (50.08%) compounds have a probability of less than 20% to decrease the MMP, and the proportion is higher in the approved drugs (73.16%). Approximately 20% of the LANaPDB (19.44%) and UNPDB (18.52%) compounds have a probability of decreasing the MMP by 20% to 40%. Therefore, most compounds in the three databases have a predicted low probability of decreasing the MMP, which indicates a low chance of causing mitochondrial dysfunction. Regardless, there have been reported several approved drugs from diverse drug groups which cause mitochondrial dysfunction and their respective mechanisms of mitochondrial impairment [137], and taking into account this knowledge during the drug discovery process can lead to reducing the probability of mitochondrial dysfunction.

##### 3.2.5.14 p53

The p53 protein is a sequence‐specific DNA‐binding transcription factor that, in response to stressful stimuli, regulates gene expression related to multiple cellular functions including, but not limited to, cell cycle arrest, cell apoptosis, cell growth, DNA repair, cell metabolism, and the immune response [139]. Practically any stress signal, whether extrinsic or intrinsic to the cell, can activate p53, so it is not surprising that the metabolic responses to limited nutrient, energy, or oxygen availability can involve p53 activation [140]. p53 is held inactive until induced by unusual, sporadic or severe stress, such as acute genotoxic stress or oncogene activation [141]. The results revealed that most of the compounds in LANaPDB (70.84%), UNPDB (69.99%), and small‐molecule approved drugs (87.55%) have a predicted probability of less than 20% to activate p53. Therefore, it is expected that most compounds in the three databases have a low probability of causing cellular stress that leads to p53 activation.

## 4 Conclusions

Herein, we report a significant update of LANaPDB, adding more than 1,000 compounds, notably adding NPs from Argentina, a country not previously present in the Latin American collection. Additionally, more than 100 compounds from Panama were added. The current version of the open‐access NP database contains 14,742 nonduplicate compounds. An *in silico* profiling of forty‐one ADMET‐related parameters of the 14,742 compounds in LANaPDB with a novel AI tool revealed that the ADMET profile is, overall, similar to the reference collections used in this work, namely, a diverse collection of NPs (UNPDB), and a set of drugs approved for clinical use. The similarity of the ADMET profile of the two NP databases to the approved drugs can be attributed to the high prevalence of NPs and NP derivatives in the approved small‐molecule drugs [1].

The following conclusions about the ADMET parameters apply to the three databases since the distribution of the results of the forty‐one ADMET‐related parameters is very similar for the three databases. The predicted absorption profile indicates that most of the compounds have a high bioavailability which is in accordance with the high probability of Caco‐2 and PAMPA permeability results. Nonetheless, the compounds presented poor aqueous solubility. Regardless, there are reported many techniques to improve the aqueous solubility [67]. The LogD_7.4_ values are in general inside the desired range between 1 and 3, a range that is expected to correspond to a favorable pharmacokinetic profile [57]. Therefore, it is expected to have a favorable pharmacokinetic profile considering the lipophilicity of the compounds. A lesser proportion has LogD_7.4_ values higher than three, whose compounds are more suitable for the nasal [73] and intramuscular [74] administration routes, which are preferred for highly lipophilic compounds. Moreover, highly lipophilic compounds can be orally absorbed via active [75] and paracellular transport [76, 77]. Nonetheless, take into account that high lipophilic compounds have a longer retention and duration of action in nontarget peripheral organs, causing considerable side effects [86].

The predicted distribution profile results show that most compounds tend to remain in tissue compartments rather than in plasma according to the volume of distribution at steady state results. Moreover, high PPBR was observed in most compounds. Besides, most compounds are predicted with high BBB penetration, beneficial for compounds that are intended to target the CNS, but with the risk of presenting side effects derived from the interaction with the CNS [87].

The predicted metabolism profile results suggest that most compounds are substrate of CYP3A4 which is responsible for metabolizing 30–50% of clinically used drugs [89]. Moreover, most compounds are predicted as noninhibitors of the CYP isozymes 1A2, 2C19, 2C9, 2D6, and 3A4 which are responsible for the metabolism of approximately two‐thirds of known drugs [88].

The predicted excretion profile indicates that most compounds have a half‐life of 12 or fewer hours, which usually corresponds to an oral dosing regimen of more than one administration per day [96]. Besides, the NPs hepatic clearance is higher than that of the approved drugs which indicates that the NPs are more susceptible to hepatic metabolism than the approved drugs.

The predicted toxicity profile showed that the three datasets present hERG blocking and no blocking compounds. Therefore, structural modifications already reported [105] should be considered to decrease the hERG affinity and thus decrease the risk of cardiac arrhythmias in those compounds which would be required. Besides, the probability of genotoxicity is low according to the mutagenicity (Ames) and ATAD5 results. Moreover, most compounds have a low probability of presenting liver injury and carcinogenicity. According to the results of the probability of activation of the androgen and estrogen receptor alpha, PPAR‐*γ*, and aromatase inhibition, most compounds have a low probability of causing reproductive dysfunction or interfering in the normal development of the reproductive organs. The acute toxicity (LD_50_) results indicate that approximately half of the compounds are slightly toxic and more than one‐third moderately toxic according to the US EPA classification. The skin sensitization results showed that approximately half of the UNPDB and approved drugs have a low probability of inducing this immune‐mediated skin reaction. Moreover, some LANaPDB compounds have a moderate probability of inducing skin sensitization. Therefore, the compounds with high probability of inducing skin sensitization should be avoided for topical pharmaceutical formulations and cosmetics. Another option is the elimination of the electrophilic moiety that allows them to be haptens, which are responsible for triggering the skin sensitization process. Nonetheless, it should be considered that some nonelectrophilic compounds (pre‐haptens and pro‐haptens) may be converted into electrophilic haptens [123]. In addition, most compounds have a low probability of having adverse effects mediated by the AHR. The observed low probability of Nrf2 activation for most compounds suggests a low likelihood of oxidative stress stimulation. Besides, the low probability of activation of the HSFs for most compounds is an indicator of a low probability of causing proteotoxic stress. Moreover, most compounds presented a low probability of causing mitochondrial dysfunction according to the observed low probability of MMP decrease. Finally, most compounds presented a low probability of p53 activation which is activated practically by any stress signal [140].

Overall, the extensive *in silico* ADMET profiling of the NP collections further supports the investigation and development of NPs, including those in the current and most updated version of LANaPDB, for hit identification in drug discovery projects.

## Supporting Information

Additional supporting information can be found online in the Supporting Information Section. **Supporting Table S1:** Box plot statistics for absorption parameters. **Supporting Table S2:** Box plot statistics for distribution parameters. **Supporting Table S3:** Box plot statistics for metabolism parameters. **Supporting Table S4:** Box plot statistics for excretion parameters. **Supporting Table S5:** Box plot statistics for toxicity parameters.

## Author Contributions

**Alejandro Gómez‐García**: conceptualization, data curation, methodology, software, validation, formal analysis, investigation, visualization, writing – original draft, writing – review & editing. **Martin J. Lavecchia**: writing – review & editing. **Dionisio A. Olmedo**: writing – review & editing. **Pablo N. Solís**: writing – review & editing. **José L. Medina-Franco**: conceptualization, formal analysis, investigation, resources, writing – review & editing, supervision, project administration, funding acquisition.

## Conflicts of Interest

The authors declare no conflicts of interest.

## Supporting information

Supplementary Material

## Data Availability

The third version of LANaPDB, the database curation code, and the code to calculate the 41 ADMET‐related parameters are available in ZENDO: https://doi.org/10.5281/zenodo.15595030. The boxplot parameters of the individual ADMET properties are included in a file in the Supporting Information.
