# Supplementary material for: Update and ADMET Profile of the Latin American Natural Product Database: LANaPDB
Source: Mol Inform. 2025 Dec 10;44(11-12):e70013. doi: 10.1002/minf.70013 (PMC12694011; doi:10.1002/minf.70013)
Supplement: Supplementary file 1 — Supplementary Material [file MINF-44-e70013-s001.pdf]

Supplementary material

Update and ADMET profile of the Latin American Natural Product Database: LANaPDB

Alejandro Gómez-García[1], Martin J. Lavecchia[2], Dionisio A. Olmedo[3], Pablo N. Solís[3], José L. Medina-Franco[1*]

[1] DIFACQUIM Research Group, Department of Pharmacy, School of Chemistry, Universidad Nacional Autónoma de México, Avenida Universidad 3000, Mexico City 04510, Mexico
[2] CEQUINOR (UNLP-CONICET, CCT La Plata, associated with CIC PBA), Departamento de Química, Facultad de Ciencias Exactas, Universidad Nacional de la Plata, La Plata B1900, Argentina
[3] Center for Pharmacognostic Research on Panamanian Flora (CIFLORPAN), College of Pharmacy, University of Panama, Av. Manuel E. Batista and Jose De Fabrega, Panama City 3366, Panama.

alex.go.ga21@hotmail.com (A.G.G.); lavecchia@quimica.unlp.edu.ar (M.J.L.); antonioagudo1960@gmail.com (D.A.O); pablonsolis@gmail.com (P.N.S.); medinajl@unam.mx (J.L.M.F.)
* Correspondence: medinajl@unam.mx; Tel. +52-55-5622-3899

Contents

		Page
**Table S1**	Box plot statistics for absorption parameters.	S2
**Table S2**	Box plot statistics for distribution parameters.	S3
**Table S3**	Box plot statistics for metabolism parameters.	S4
**Table S4**	Box plot statistics for excretion parameters.	S5
**Table S5**	Box plot statistics for toxicity parameters.	S6

Table S1. Box plot statistics for absorption parameters.

Parameter	Metric	Approved drugs	LANaPDB	UNPDB	Units
Human intestinal absorption	Max	100	100	100	%
	Q3	99.97	99.99	99.97	
	Median	99.72	99.91	99.68	
	Q1	96.73	99.23	90.41	
	Min	92.20	98.09	76.07	
Oral bioavailability	Max	0.99	0.99	1	*
	Q3	0.91	0.83	0.79	
	Median	0.82	0.72	0.64	
	Q1	0.65	0.57	0.44	
	Min	0.27	0.17	0.02	
Caco-2 cell effective permeability	Max	1.27	-0.25	0.59	log(10-6 cm/s)
	Q3	-1.91	-3.13	-2.98	
	Median	-3.08	-4.03	-4.13	
	Q1	-4.29	-5.05	-5.36	
	Min	-7.71	-7.92	-8.90	
PAMPA permeability	Max	4.96	5.89	6.16	%
	Q3	2.77	3.24	3.32	
	Median	1.51	2.30	2.25	
	Q1	0.03	1.46	1.13	
	Min	-3.56	-1.19	-2.16	
Aqueous solubility	Max	0.78	1.30	2.33	log(mol/L)
	Q3	-7.11	-5.45	-5.94	
	Median	-9.80	-7.81	-8.70	
	Q1	-12.38	-9.96	-11.45	
	Min	-20.23	-16.72	-19.71	
Hydration free energy	Max	-3.62	-3.63	-3.42	kcal/mol
	Q3	-4.56	-4.55	-4.67	
	Median	-4.94	-4.86	-5.05	
	Q1	-5.44	-5.19	-5.66	
	Min	-6.75	-6.13	-7.14	
Lipophilicity (log$D_{7.4}$)	Max	99.93	99.86	99.96	log-ratio
	Q3	94.79	96.53	94.82	
	Median	80.10	90.40	81.65	
	Q1	37.65	76.58	43.63	
	Min	0.09	46.76	0.07	
P-glycoprotein inhibition	Max	99.42	99.91	99.96	%
	Q3	46.96	63.38	73.41	
	Median	11.35	33.34	35.77	
	Q1	1.35	12.93	10.78	
	Min	0	0.01	0	

*This parameter is unitless.

Table S2. Box plot statistics for distribution parameters.

Parameter	Metric	Approved drugs	LANaPDB	UNPDB	Units
Volume of distribution at steady state	Max	100	99.92	100	L/kg
	Q3	95.64	91.92	85.85	
	Median	73.40	78.01	58.47	
	Q1	38.92	53.03	24.99	
	Min	1.46	0.88	0.56	
Plasma protein binding rate	Max	115.93	122.40	129.19	%
	Q3	90.76	93.92	97.62	
	Median	76.17	84.48	87.72	
	Q1	56.27	74.72	75.43	
	Min	5.45	46.01	42.14	
Blood-brain barrier penetration	Max	14.01	15.16	16.45	%
	Q3	4.80	5.76	5.47	
	Median	1.31	2.56	1.74	
	Q1	-1.39	-0.54	-1.84	
	Min	-9.94	-9.89	-12.82	

Table S3. Box plot statistics for metabolism parameters.

Parameter	Metric	Approved drugs	LANaPDB	UNPDB	Units
CYP1A2 inhibition	Max	81.24	75.25	80.31	%
	Q3	32.93	30.43	32.34	
	Median	5.46	2.62	2.42	
	Q1	0.67	0.52	0.36	
	Min	0	0	0	
CYP2C19 inhibition	Max	81.62	98.58	92.56	%
	Q3	34.49	43.01	39.09	
	Median	11.47	17.11	11.93	
	Q1	2.55	5.96	3.45	
	Min	0.01	0.02	0	
CYP2C9 inhibition	Max	47.81	54.19	59.98	%
	Q3	19.69	23.08	25.02	
	Median	4.12	5.97	5.67	
	Q1	0.86	2.33	1.71	
	Min	0	0	0	
CYP2D6 inhibition	Max	67.50	37.27	31.66	%
	Q3	27.70	15.99	13.64	
	Median	5.06	5.32	4.66	
	Q1	1.08	1.80	1.62	
	Min	0	0	0	
CYP3A4 inhibition	Max	88.52	99.66	99.87	%
	Q3	36.23	56.75	46.56	
	Median	8.79	20.80	14.80	
	Q1	0.80	4.94	2.91	
	Min	0	0	0	
CYP2C9 substrate	Max	63.36	41.26	41.18	%
	Q3	30.40	18.78	18.46	
	Median	17.12	9.27	9.01	
	Q1	8.06	3.78	3.30	
	Min	0.02	0.18	0.02	
CYP2D6 substrate	Max	80.38	54.17	45.78	%
	Q3	34.49	24.15	20.13	
	Median	11.38	10.44	7.90	
	Q1	3.72	4.11	3.03	
	Min	0.24	0.16	0.08	
CYP3A4 substrate	Max	95.34	96.91	96.77	%
	Q3	71.48	77.10	75.77	
	Median	51.30	67.60	62.32	
	Q1	26.38	53.99	46.23	
	Min	0.39	19.34	1.91	

Table S4. Box plot statistics for excretion parameters.

Parameter	Metric	Approved drugs	LANaPDB	UNPDB	Units
Half-life	Max	75.16	65	89.93	hours
	Q3	26.56	21.73	33.25	
	Median	6	5.99	11.12	
	Q1	-6.51	-7.21	-4.55	
	Min	-51.63	-50.48	-61.19	
Drug clearance (hepatocyte)	Max	154.07	148.58	162.90	uL/min/106 cells
	Q3	71.50	93.58	91.39	
	Median	39.99	76.32	67.87	
	Q1	15.63	56.64	43.68	
	Min	-65.28	1.28	-27.84	
Drug clearance (microsome)	Max	109.16	128.70	142.11	uL/min/mg
	Q3	44.36	63.41	67.78	
	Median	17.49	41.03	41.57	
	Q1	-0.76	19.71	18.21	
	Min	-55.76	-42.29	-56.13	

Table S5. Box plot statistics for toxicity parameters.

Parameter	Metric	Approved drugs	LANaPDB	UNPDB	Units
hERG blocking	Max	99.69	97.51	98.93	%
	Q3	76.18	61.53	68.07	
	Median	30.69	37.33	45.62	
	Q1	8.45	18.36	21.16	
	Min	0.04	0.03	0.02	
Clinical toxicity	Max	68.51	48.74	37.65	%
	Q3	29.45	22.05	17.52	
	Median	9.24	11.41	9.32	
	Q1	3.28	4.26	4.10	
	Min	0	0	0	
Mutagenicity	Max	75.58	97.78	90.10	%
	Q3	34.29	48.80	43.15	
	Median	16.31	28.30	25.03	
	Q1	6.24	13.23	11.85	
	Min	0.04	0.06	0.01	
Drug induced liver injury	Max	99.77	99.75	99.83	log(1/(mol/kg))
	Q3	83.67	65.38	70.49	
	Median	42.31	35.27	34.35	
	Q1	13.16	16.02	14.14	
	Min	0.04	0.11	0.05	
Carcinogenicity	Max	70.82	22.50	30.89	%
	Q3	31.76	10.35	13.70	
	Median	14.26	4.80	5.67	
	Q1	5.59	2.24	2.24	
	Min	0.10	0.04	0.01	
Acute toxicity (letal dose 50)	Max	4.15	4.78	4.94	%
	Q3	2.92	3.33	3.31	
	Median	2.52	2.81	2.73	
	Q1	2.10	2.35	2.22	
	Min	0.92	0.93	0.59	
Skin reaction	Max	99.82	98.86	99.77	%
	Q3	69.52	63.06	63.18	
	Median	41.75	46.31	41.47	
	Q1	22.84	30.50	25.47	
	Min	3.47	3.60	2.21	
Androgen receptor (full length)	Max	13.31	50.16	28.82	%
	Q3	6.09	21.91	13.04	
	Median	2.82	7.57	5.63	
	Q1	1.20	3.05	2.51	
	Min	0	0.02	0	
Androgen Receptor (Ligand Binding Domain)	Max	6.46	29.27	20.55	%
	Q3	2.81	12.46	8.95	

	Median	1.08	4.33	3.67	
	Q1	0.34	1.25	1.21	
	Min	0	0	0	
Aryl hydrocarbon receptor	Max	31	46.88	41.96	%
	Q3	12.84	19.29	17.21	
	Median	2.85	2.28	2.29	
	Q1	0.72	0.90	0.72	
	Min	0	0.01	0	
Aromatase	Max	26.21	53.77	46.92	%
	Q3	10.73	25.02	21.07	
	Median	2.66	13.23	9.79	
	Q1	0.38	5.84	3.83	
	Min	0	0	0	
Estrogen receptor (full length)	Max	37.22	51.49	56.63	%
	Q3	17.91	26.76	28.47	
	Median	8.87	17.04	17.37	
	Q1	5.03	10.26	9.69	
	Min	0.10	0.53	0.04	
Estrogen receptor (ligand binding domain)	Max	10.33	26.61	33.05	%
	Q3	4.57	12.39	14.89	
	Median	1.74	5.81	6.89	
	Q1	0.62	2.90	2.79	
	Min	0	0.01	0	
Peroxisome proliferator-activated receptor gamma	Max	6.04	11.40	13.77	%
	Q3	2.49	5.02	5.90	
	Median	0.45	2.04	1.91	
	Q1	0.11	0.76	0.66	
	Min	0	0	0	
Nuclear factor (erythroid-derived 2)-like 2 / Antioxidant responsive element	Max	69.14	99.51	99.94	%
	Q3	30	49.23	47.15	
	Median	12.15	28.72	25.22	
	Q1	3.84	13.86	11.86	
	Min	0.01	0.01	0	
ATPase family AAA domain-containing protein 5 (ATAD5)	Max	7.03	18.93	21.30	%
	Q3	2.89	7.99	8.85	
	Median	0.56	2.88	2.68	
	Q1	0.12	0.70	0.54	
	Min	0	0	0	
Heat shock factor response element	Max	11.21	31.44	37.54	%
	Q3	4.76	13.83	15.98	
	Median	1.27	5.49	5.26	
	Q1	0.42	2.07	1.6	
	Min	0	0	0	
Mitochondrial membrane potential	Max	55.83	99.72	99.77	%
	Q3	22.71	48.3	50.58	

	Median	4.19	19.87	19.93	
	Q1	0.58	7.49	5.55	
	Min	0	0	0	
	Max	19.76	52.85	56.38	
	Q3	8.29	23.1	24.27	
Tumor protein p53	Median	1.91	9.59	9.59	%
	Q1	0.39	3.24	2.86	
	Min	0	0	0	
